# Maternal and Neonatal Mortality in South-West Ethiopia: Estimates and Socio-Economic Inequality

**DOI:** 10.1371/journal.pone.0096294

**Published:** 2014-04-30

**Authors:** Yaliso Yaya, Kristiane Tislevoll Eide, Ole Frithjof Norheim, Bernt Lindtjørn

**Affiliations:** 1 Centre for International Health, Faculty of Medicine and Dentistry, University of Bergen, Bergen, Norway; 2 Department of Global Public Health and Primary Care, Faculty of Medicine and Dentistry, University of Bergen, Bergen, Norway; 3 Arba Minch College of Health Sciences, Arba Minch, Ethiopia; Baylor College of Medicine, United States of America

## Abstract

**Introduction:**

Ethiopia has achieved the fourth Millennium Development Goal by reducing under 5 mortality. Nevertheless, there are challenges in reducing maternal and neonatal mortality. The aim of this study was to estimate maternal and neonatal mortality and the socio-economic inequalities of these mortalities in rural south-west Ethiopia.

**Methods:**

We visited and enumerated all households but collected data from those that reported pregnancy and birth outcomes in the last five years in 15 of the 30 rural kebeles in Bonke woreda, Gamo Gofa, south-west Ethiopia. The primary outcomes were maternal and neonatal mortality and a secondary outcome was the rate of institutional delivery.

**Results:**

We found 11,762 births in 6572 households; 11,536 live and 226 stillbirths. There were 49 maternal deaths; yielding a maternal mortality ratio of 425 per 100,000 live births (95% CI:318–556). The poorest households had greater MMR compared to richest (550 vs 239 per 100,000 live births). However, the socio-economic factors examined did not have statistically significant association with maternal mortality. There were 308 neonatal deaths; resulting in a neonatal mortality ratio of 27 per 1000 live births (95% CI: 24–30). Neonatal mortality was greater in households in the poorest quartile compared to the richest; adjusted OR (AOR): 2.62 (95% CI: 1.65–4.15), headed by illiterates compared to better educated; AOR: 3.54 (95% CI: 1.11–11.30), far from road (≥6 km) compared to within 5 km; AOR: 2.40 (95% CI: 1.56–3.69), that had three or more births in five years compared to two or less; AOR: 3.22 (95% CI: 2.45–4.22). Households with maternal mortality had an increased risk of stillbirths; OR: 11.6 (95% CI: 6.00–22.7), and neonatal deaths; OR: 7.2 (95% CI: 3.6–14.3). Institutional delivery was only 3.7%.

**Conclusion:**

High mortality with socio-economic inequality and low institutional delivery highlight the importance of strengthening obstetric interventions in rural south-west Ethiopia.

## Introduction

In 2010, there were 287,000 maternal deaths in the world from causes of pregnancy related complications, which is 50% down from the 1990 baseline [1]. Every year, four million newborns die during the neonatal period (28 days after birth) [2], while 3.2 million pregnancies end with stillbirths [3]. Among these deaths, 99% of the neonatal deaths and stillbirths as well as 98% of maternal deaths, occur in low- and middle-income countries. Moreover, 40% of global, and 29% of African child mortality is caused by neonatal deaths [4]. The Millennium Development Goals (MDG-4 and 5) aim to reduce child mortality by two-thirds, and maternal mortality by three-quarters, between 1990 and 2015. High maternal and neonatal deaths and stillbirths often occur because of inadequate care during pregnancy and childbirth. Accordingly, these deaths are considered sensitive indicators of the quality of a healthcare system in an area [5]. High neonatal deaths and stillbirths are often related to maternal health; complications during pregnancy, childbirth, and the post-natal period increase the risk of death for both the baby and the mother [6].

Ethiopia has achieved MDG-4 by reducing two-thirds of under 5 mortality per 1000 live births from 204 in 1990 to 68 in 2012 [7]. Ethiopia's achievement has been attributed to the country's community-based health promotion and disease prevention programme through a health extension package [8]. However in terms of neonatal mortality, maternal mortality, and stillbirth reduction, challenges still remain. Ethiopia is among 10 countries that contributed to two-thirds of global neonatal deaths in 2005 [2] and stillbirths in 2009 [9]. UN agencies (WHO and UNFPA) and World Bank data also indicate a decline in the maternal mortality ratio (MMR) per 100,000 live births in Ethiopia from 950 in 1990 to 350 in 2011 [1]. Nevertheless, there are often controversies over these estimates. For example, the 2011 national Demographic and Health Survey (DHS) reported an MMR of 676 per 100,000 live births, which is close to two-folds higher compared to the UN estimate [10]. According to the 2007 National Census, over 84% of the estimated 90 million people in Ethiopia live in rural areas with limited access to quality health care[11]. In 2008, a nationwide study showed that 7% of all deliveries took place at health institutions while only 3% in facilities that could provide comprehensive essential obstetric care [12].

In places such as rural Ethiopia, where there is no birth and death registration and the majority of births and deaths take place outside of health institutions, measuring maternal and neonatal mortality is difficult [13]. In some places, methods such as demographic surveillance systems help to find and measure maternal and neonatal mortalities [14]. Unfortunately, this alternative method does not exist in the Gamo Gofa province of Ethiopia. Maternal mortality can also be estimated through low-cost innovative options of the sisterhood method, which asks adult siblings about their sisters' death related to pregnancy or childbirth during reproductive age [15]. Nevertheless, results from the sisterhood method refer to many years before the survey and may not show the current magnitude of the problem [16]. Findings from well-planned household surveys that use large samples in high fertility and high mortality areas can be useful in providing real-time data to motivate actions [17]. The aim of this study was to estimate maternal and neonatal mortality, the stillbirth rate, the institutional delivery rate, and household risk factors associated with these mortality outcomes in rural south-west Ethiopia.

## Methods and Materials

### Ethics statement

The Ethical Review Committee for the Health Research of Southern Nations Nationalities and Peoples' Regional State (SNNPRS) Health Bureau in Ethiopia, and the Regional Committee for Health Research Ethics of North Norway (REK Nord) approved the study. We obtained informed verbal consent from all respondents and the response was recorded on the questionnaire as “accepted” or “declined” to participate. Almost all approached households were willing to be interviewed, and written consent was not considered because a large number of the respondents were illiterates. The study involved only interview and the ethics committee approved the verbal consent procedure. Additionally, minors were not included in this study.

### Definitions

#### Verbal autopsy for maternal deaths

A method for finding out the medical causes of death and ascertaining the factors that may have contributed to the death in women who died outside of a medical facility. It consists of interviewing people (family members, neighbours, traditional birth attendants) who had knowledge about the events leading to the death [18].

#### Neonatal mortality

A death within 28 days of an alive born baby.

#### Maternal mortality

A death of a woman while pregnant, in labour, or within 42 days of the termination of pregnancy, irrespective of the duration and site of the pregnancy, from any cause related to or aggravated by the pregnancy or its management, but not from accidental or incidental causes (ICD-10) [19].

#### Stillbirth

A birth of a dead fetus after 28 weeks of gestation. We did not use the baby-weight criteria of classifying stillbirths, as it was not possible to measure weight in the rural area.

#### Household

A person or a group of people living in a room or rooms and sharing common things together. In cases of polygamy (more than one wife for a man, we considered each wife as a separate household as they culturally have separate houses.

#### Maternal mortality ratio (MMR)

Is the number of maternal deaths in a population during a given time period per 100,000 live births during the same period.

#### Neonatal mortality ratio (NMR)

Is the number of newborn deaths (within 0–28 days) in a population per 1,000 live births in the same population.

#### Stillbirth rate

Is the number of births of dead fetuses after 28 weeks of gestation per 1,000 births.

### Study area

We conducted this study in Bonke, one of the 15 woredas in the Gamo Gofa zone in south-west Ethiopia. In 2010, the woreda had a population of 173,240 people [11]. A kebele is the lowest administrative structure with 5,000 to 7,000 residents in the Ethiopian government system. Bonke has 31 kebeles and one of these, the administrative centre, has a town status with a population of 6,347 people in 2007. Table 1 shows the profile of the 15 kebeles included in this study. Bonke is 618 km from Addis Ababa, and 68 km from Arba Minch (zonal capital) where the nearest hospital is situated. Nevertheless, over half of the remote areas of Bonke are more than 100 km (20 hours walking distance) away from the hospital in Arba Minch. An estimated three-fourths of the population also live in villages far from the motorable road (≥6 km). The only road to the woreda is the road from Arba Minch to Kamba, which crosses parts of Bonke. Overflowing rivers during the rainy season often interrupt the road. So, often people have to carry critical patients or use transport animals such as horses and mules to go to the hospital.

**Table 1:** Background information comparing study population with national census data.

	Bonke 2010(current study)	Census 2007 [16] (adjusted to 2010)
Number of households in 15 Kebeles	11 920	12 681
Population of the 15 Kebeles	72 712§	78 181
Average persons per household	6.14	6.17
Crude birth rate (per 100 population)	3.22	3.60†
Percent of under-5 year population	15	16μ
Percent of illiterate adults (≥15 years)	58. 5^A^	64.5^B^

§ =  obtained by multiplying average persons per household in studied households (6.1) with the total households counted during the study (11 920).

† =  DHS 2011 for rural Ethiopia (no separate data for Bonke) [10].

μ =  rural Bonke.

A =  Heads of interview households, Bonke, 2010.

B =  Adult men (≥15 years of age) Gamo Gofa zone (rural).

Health care is provided by a health centre in the town (Geresse) as well as three other rural health centres. There are no medical doctors working in the woreda; a few health officers (people with a bachelor's degree in medical training), nurses, and midwives staff the health centres. In Bonke, there is no access to lifesaving comprehensive essential obstetric care that can provide caesarean sections, blood transfusions, and effective care to sick and low birth-weight newborns. This study was part of an implementation project to reduce maternal mortality in Gamo Gofa. The project trains health officers in emergency obstetric services, community health workers in identifying and referring high-risk mothers, in addition to equipping health centres and hospitals with essential instruments.

### Study design and period

The study was a cross-sectional household survey with a five-year recall of events prior to the data collection. We collected the data in February 2011 from all households that had births and pregnancy outcomes between January 2006 and December 2010. We purposely selected January 2006 as the starting reference period for the recall because it was the immediate period after the 2005 National Election, an event well known to all respondents.

### Sampling

We based our sample size calculation on the assumptions of a crude national birth rate of 35 per 1,000 population, and a neonatal mortality ratio of 35 per 1,000 live births [20]. We aimed to detect a minimum of 350 neonatal deaths to make empirical estimates and assess the household risk factors associated with neonatal deaths. To find 350 neonatal deaths we needed to find at least 10,000 live births in five years (an average of 2,000 per year) within the population. With a fertility rate of 35 births per 1,000 people, 2,000 live births per year could be obtained from an estimated population of 57,143. Assuming a constant birth rate over the five years, we projected the population of 57,143 in 2006 to be 67,244 in 2010 (half the rural population in Bonke).

We used OpenEpi, open source calculator (www.openepi.com), and calculated the minimum sample needed based on information that three-fourths of the study households resided far from the motorable road (≥6 km), thereby expecting a neonatal mortality prevalence twice that among households far from the road. We used a statistical power of 80%, a 95% confidence interval, and an assumption that 4% of households far from motorable road could experience neonatal mortality to calculate the number of households needed for the study. This provided 5,187 households with expected births in the five years before the survey. On average, we expected two births per household over five years yielding 10,374 births. The neonatal mortality rate from the estimated number of households was also assumed to provide enough power to detect other risk factors (wealth, education, non-spaced births).

We also assumed that a number of maternal deaths among the estimated 10,374 births would give an optimum MMR estimate, and a similar assumption was applied for stillbirths. Taking into account a potential 10% of non-responders, we decided to study 50% of the rural population in the Bonke woreda and we randomly selected 15 of the 30 rural kebeles in Bonke. Data collectors visited all of the households in the selected 15 kebeles asking about any pregnancy and birth outcomes (abortion, alive and stillbirths, neonatal and maternal deaths) in the households over the previous five years. Enumerators noted the number of households in each kebele, collecting data from the households that had pregnancy and birth outcomes during the stated time period. As projected from the Ethiopian 2007 census [11], the selected kebeles had a population of 78,181 people in 2010.

### Variables and questions

The primary outcome variables were maternal and neonatal mortality and stillbirth rates while a secondary outcome variable was rate of skilled delivery service utilization. We also used the following household predictor variables: household wealth (assets index), educational level of heads of households, the number of births in a household over five years, and household distance to a motorable road.

Survey questions included: where each delivery took place (home, health post, health centre, and hospital) and who attended the birth (family member, community health extension worker, skilled health professional-doctor, midwife). We also asked about the place where the maternal death occurred (at home or within a health facility) as well as what had happened to the fetus (stillbirth, neonatal death, or alive at the time of data collection). Respondents were asked about whether the newborn was alive or dead at the end of the 4th week after delivery. If the response was “dead”, then we asked about the timing of death (in weeks) in relation to the birth. However, we did not investigate the causes of deaths for neonatal deaths and stillbirths, assuming it would be difficult for rural respondents to answer it properly. In the households that had deaths of women -in reproductive age (15–49 years), we used questions modified from the WHO manual for verbal autopsy for maternal death to investigate the causes of deaths [18].

The questions included: whether the mother was pregnant, in the process of giving birth, or in postnatal period after birth, what main medical condition or symptom was associated with her death, what assistance she received, and from whom she received help. A nurse decided on pre-coded choices of the major causes of maternal deaths (bleeding, prolonged labour, fever and convulsions, including the option of “others”) based on quick algorithmic analysis of information provided by the respondents. Sensitive questions related to abortion deaths were placed at the end of the interview to minimize the intentional hiding of information. We collected information on the estimated walking distance (in hours) from each house to the nearest health centre, the nearest motorable road, and the nearest hospital. Based on the local experience of one hour of walking time per 5 km for an average person, we converted the walking distance into kilometers.

### Data collection

We recruited 15 natives from the respective study villages who had completed the 12^th^ grade for data collection. The purpose of selecting data collectors from their respective kebele of data collection was to reduce the potential recall bias by the respondents. Data collectors are aware of many vital events in the villages they collected data by living and participating in social events such as birth celebrations, mourning rituals, and burials at the time of the deaths. Five diploma graduates who had a thorough knowledge of the culture and language of the area supervised the data collection.

The data collectors were trained for two days on pre-testing field interviews, translating the questions from “Amharic” (the official Ethiopian state language) to “Gamotho” (the language of the ethnic “Gamo” community) and how to introduce the simplified verbal autopsy questions. Depending whoever was present at home during the visit, the respondent was the father or mother for a recently deceased newborn. In cases of death of a married woman, we interviewed a husband while in the absence of a husband, an adult relative or an adult child of the deceased was interviewed. For those who were unmarried, we asked parents or siblings. If the respondents were not present at home during the first visit, the data collectors re-visited the next day in the early morning. Less than 1% of households were missed after two visits.

### The wealth-index creation

For the wealth index, we selected 10 variables of household assets with the highest standard deviation (>0.20), as recommended by Seema Vyas and colleagues [21]. The types of asset variables and their standard deviations are presented in Table S1 in File S1. We transformed the categorical variables into dichotomous (0–1) indicators: 0 for indicators of poor wealth and 1 for indicators of good wealth. We examined the dichotomous variables by using the principal component analysis (PCA) to produce a factor score for each household with households being assigned a rank according to the factor score. Because of the low number of maternal deaths in the socio-economic classes for calculation, we divided households into four equal categories (quartiles), rather than the widely used five classes. Each category was comprised of 25% of the households studied. Table S2 in File S1 shows the mean score, standard deviations, communalities, and correlations of the variables to the first (main) component. The total variance explained by the first component was 20.58%, with an eigenvalue of 2.06.

### Data analysis

We used two units of analysis (household and birth). By using births as the unit of analysis, we presented descriptive tabulations of outcomes in the form of rates and ratios. By using the household as a unit of analysis, and applying logistic regression, we present household risk factors associated with the mortality outcomes (Tables 2, 3, and 4). We used SPSS 16 (Statistical Package for Social Sciences) for the data entry and analysis [22]. Data are freely available from the corresponding author on request.

**Table 2:** Socio-demographic backgrounds of household-survey participants, Bonke, Gamo Gofa, south-west Ethiopia, 2011.

Variables	Category	number	%
Visited households	With pregnancy and birth outcomes^A^	6572	55
	Without pregnancy and birth outcomes	5348	45
	Total (visited HHs)	11920	100
Births	Live births	11536	98
	Still births	226	2
	Total (all births)	11762	100
Households with events	Stillbirths	192	3
	Neonatal deaths	265	4
	Maternal deaths	49	0.7
	Without events	6066	92.3
	Total (HHs)	6572	100
Education of heads of	Illiterate (cannot read/write)	3842	58.5
households	Primary (1–8th)	2446	37.2
	Higher (9th +)	279	4.2
	Missing	5	0.1
	Total (HHs)	6572	100
Occupation of heads of households	Subsistence farming	6289	95.7
	Farming and small trade	204	3.1
	Salary employed	79	1.2
	Total (HHs)	6572	100
Distances of HHs from	≤5 km	1624	24.7
motorable road	≥6 km	4948	75.3
	Total (HHs)	6572	100
Health centre distance	≤10 km^B^	3258	49.6
	≥11 km	3314	50.4
	Total (HHs)	6572	100
Place of delivery	Home (assisted by family or relative)	10861	92.3
	Health post (attended by HEWs)^C^	470	4.0
	Skilled institutions (HC and Hospital)	431	3.7
	Total (births)	11762	100
Health centre delivery vs	HC deliveries among ≤10 km from HC	274/5766	4.75
HC distance	HC deliveries among ≥11 km from HC	64/5996	1.07
	Total HC births out of all births	338/11762	2.87

Note: ^A^Households that had the outcomes over five years before the survey (focus of this study), ^B^10 km distance is Ethiopian government plan for physical access to health centres, HC  =  Health Centre, HHs  =  households, HEWs  =  ^C^Health Extension Workers (non-skilled birth attendants).

**Table 3:** Causes and places of maternal deaths, Bonke, Gamo Gofa, south-west Ethiopia, 2006–2010.

Variables		number	%
Causes of death	Fever	14	29
	Bleeding	13	27
	Prolonged labour	8	16
	Convulsion	8	16
	Others*	6	12
	Total (deaths)	49	100
Places of death	Home (community)	43	88
	Health institution	6	12
	Total (deaths)	49	100

Note: *other causes include: two abortions, two anaemia, and two cause not reported.

**Table 4:** Maternal mortality across socio-economic factors, south-west Ethiopia, 2006–2010.

Variable	Category	Maternal deaths	Live births	MMR^A^	OR (95% CI)
Wealth^B^	Richest 25%	6	2506	239	Ref
	Rich 25%	11	3146	350	1.46 (0.54, 4.28)
	Poor 25%	14	2953	474	1.98 (0.77, 5.59)
	poorest 25%	15	2725	550	2.29 (0.91, 6.44)
Education^C^	Higher (9th +)	0^D^	468	---	Ref
	Primary (1–8th)	18	4492	401	2.85 (0.42, 77.9)
	Illiterate	31	6568	472	3.35 (0.52, 89.9)
Distance to road	≤5 km	9	2754	327	Ref
	≥6 km	40	8782	455	1.40 (0.67, 2.88)
No. of births (in 5 yr)	≤2	39	8620	452	Ref
	≥3	10	2916	343	0.76 (0.38, 1.52)
Place of deaths	Home (comm.)	43	11160	385	Ref
	Health institution	6	376	1596	4.14 (1.75, 9.79)

Note: ^A^maternal mortality ratio per 100,000 live births.

B 119 households, including 3 with maternal deaths, have missing value on the wealth index due to incomplete asset variables.

C Education of head of household. ^D^zero was replaced by 0.5 during analysis to make calculation defined.

## Results

### Demographic description

Table 1 describes households and population of the study area. Data collectors enumerated all households (11, 920) in the selected 15 kebeles but collected data from 6,572 households that had pregnancy and birth outcomes in the last five years before the data collection. In the 6,572 households that had pregnancy and birth outcomes, there were 40,357 persons, an average of 6.1 persons per household.

Of the 6,572 household heads, 3,842 (58.5%) were not able to read and write (illiterate), 2,446 (37.2%) had an elementary education (grade 1–8), and 279 (4.2%) had completed 9th grade or more. Regarding the occupations of the head of the households, 6,289 (95.7%) engaged in farming, 204 (3.1%) in farming mixed with small trade, while 79 (1.2%) were salary employed ($ 25–50 per month) (Table 2).

### Description of births

Table 2 presents the backgrounds of households with pregnancy and birth outcomes and description of births. We found 11,762 births in 6572 households over 5 years (3.2% annual crude birth rate), of which 11,536 were live births. Of the 11,762 total births, 10,861 (92.3%) took place at home, 470 (4%) at health posts, 338 (2.9%) at health centres and 93 (0.8%) at hospitals. A health post is a two-room building which is the lowest ranked health facility and staffed by two community agents known as health extension workers whom received one year of general health training. Health centres are medium level institutions with health officers and nurses, and some have midwives. The overall institutional delivery rate (health centre and hospital combined) was 3.7% (431/11762).

### Health centre births

Births that occurred within a 10 km distance of health centres, were five-times more likely to use health centres for delivery, 4.75% (274/5766), compared to households ≥11 km away, 1.07% (64/5996); OR: 4.62 (95% CI: 3.51–6.09) (Table 2). A 10 km distance is the Ethiopian government plan to achieve health centre physical access to the population.

### Description of deaths

**Figure 1:**
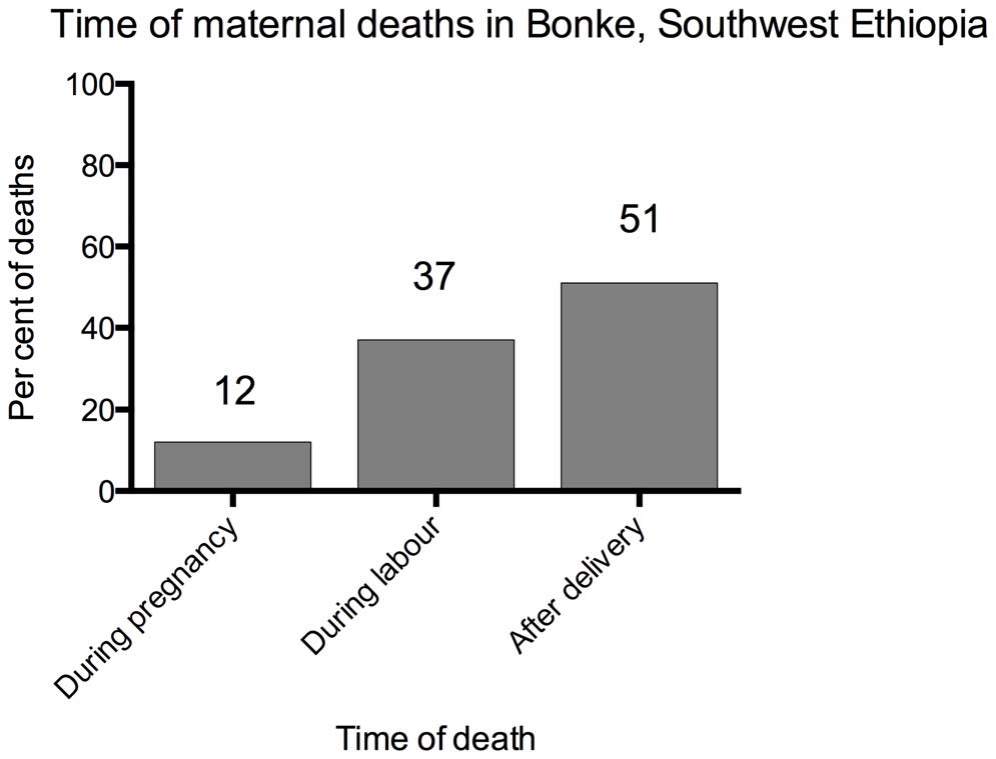
Time of maternal deaths, Bonke woreda, south-west Ethiopia, 2010.

There were 49 maternal deaths, resulting in a maternal mortality ratio (MMR) of 425 (95% CI: 318–556) per 100,000 live births. Among the 49 maternal deaths, 6 (12%) occurred during pregnancy, 18 (37%) during labour, and 25 (51%) after birth within six weeks (Figure 1). The primary causes of death were: fever 14 (29%), bleeding 13 (27%), prolonged labour 8 (16%), convulsion 8 (16%), and others 6 (12%) (Table 3). Other causes included two abortions and one anemia while three deaths were not classified. Regarding the places of maternal death, most [88% (43/49)] of the maternal deaths occurred during home deliveries, and health facilities were able to identify only 12% (6/49) of the maternal deaths found in this study (Table 3).

**Figure 2:**
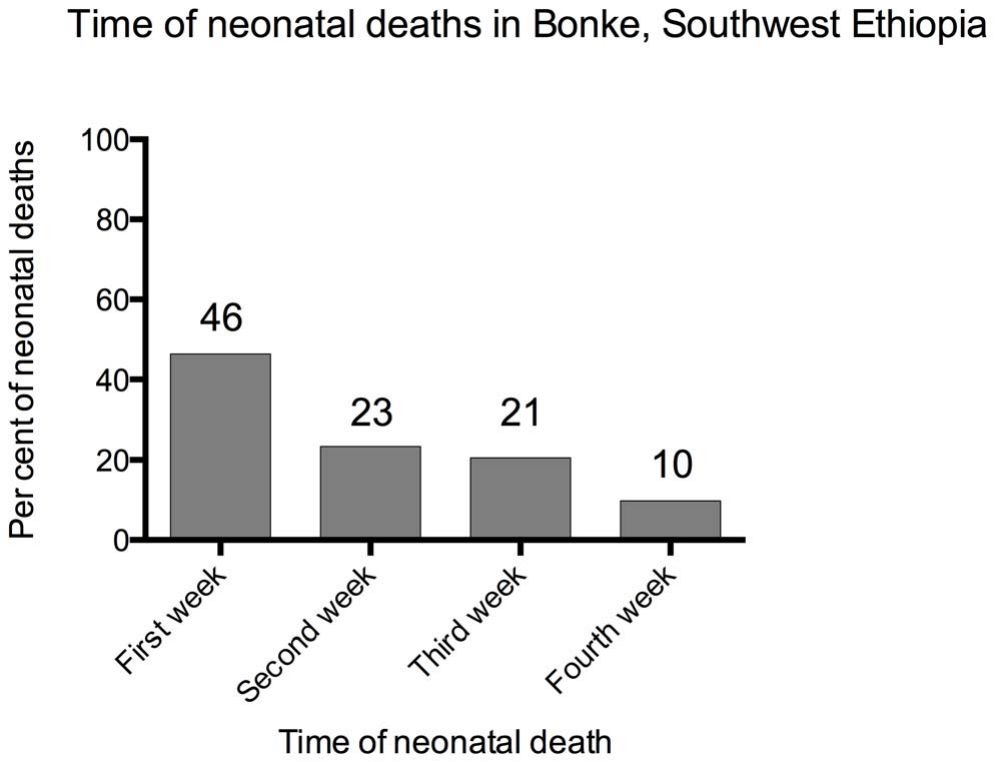
Time in weeks of neonatal deaths, Bonke woreda, south-west Ethiopia, 2010.

We found 308 neonatal deaths, which yields a neonatal mortality ratio (NMR) of 27 (95% CI: 24–30) per 1,000 live births. Out of the 308 neonatal deaths reported, 143 (46.4%) died in the first week, 72 (23.4%) in the second week, 63 (20.5%) in the third week and 30 (9.7%) in the fourth week (Figure 2). There were 226 stillbirths out of 11,762 total births yielding a stillbirth rate (SBR) of 19 (95% CI: 17–22) per 1,000 births.

### Household risk for mortality outcomes

#### Maternal mortality

Table 4 shows the MMR differences across different risk factors. The MMR was increased in households in the poorest quartile compared to the richest (550 vs 239 per 100,000 live births); OR: 2.29 (95% CI: 0.91–6.44). However, socio-economic factors examined (wealth, distance from road, education, and non-spaced births) did not have statistically significant association with maternal mortality because of the relative rarity of maternal deaths in terms of absolute numbers.

#### Neonatal mortality

Table 5 describes the household risk factors associated with neonatal mortality. Neonatal mortality was greater among the poorest quartile households compared to the richest; adjusted OR (AOR): 2.62 (95% CI: 1.65–4.15). However, the highest risk was in the wealthy class; AOR: 3.57 (95% CI:2.37–5.38). The poorest were in the second highest at risk groups. The likelihood of neonatal mortality was also increased among households with illiterate heads compared to where the heads had a higher education (9th grade or more); AOR: 3.54 (95% CI: 1.11–11.30), in households far from a motorable road (≥6 km) compared to those within 5 km of a road; AOR: 2.40 (95% CI: 1. 56–3.69), and greater among households that had three or more births in five years compared to those that had two or less births; AOR: 3.22 (95% CI: 2.45–4.22).

**Table 5:** Factors associated with neonatal mortality, south-west Ethiopia, 2006–2010.

Predictors	Category	Neonatal deaths in the household?^A^ (n = 6572 HHs)		Crude		Adjusted^E^	
		Yes	No	OR	95% CI	OR	95% CI
Wealth^B^	Richest 25%	31	1322	Ref		Ref	
	Rich 25%	124	1685	3.14	2.12, 4.74	3.57	2.37, 5.38
	Poor 25%	46	1662	1.18	0.74, 1.89	1.92	1.19, 3.10
	Poorest 25%	57	1526	1.59	1.02, 2.48	2.62	1.65, 4.15
Education^C^	Higher (9th +)	3	276	Ref		Ref	
	Primary (1–8th)	79	2367	3.07	1.08, 12.37	2.86	0.89, 9.18
	Illiterate^D^	182	3660	4.57	1.64, 18.24	3.54	1.11, 11.30
Distance to road	≤5 km	28	1596	Ref		Ref	
	≥6 km	237	4711	2.87	1.93, 4.26	2.40	1.56,3.69
No. of births^F^	≤2	170	5388	Ref		Ref	
	≥3	95	919	3.28	2.53, 4.25	3.22	2.45, 4.22

A HH =  households (because there were more than one events in some households, the number of households having neonatal deaths are different from the number of neonatal deaths; 308 neonatal deaths in 265 households).

B 119 households, including 7 with neonatal deaths, have missing value on the wealth index due to incomplete asset variables.

C Education of head of household. ^D^Illiterate  =  cannot read and write ^E^adjusted to the other variables in the table. ^F^number of births in five years.

Note: Hosmer-Lemeshow Test of Model fit: X^2^ (df) = 9.14 (7); p = 0.24. A p-value greater than 0.05 shows that the model well fit the data.

#### Stillbirths

Table 6 shows the factors associated with stillbirth which were greater in households in the poorest quartile compared to the richest; AOR: 3.13 (95% CI: 1.66–5.87). However, similar to neonatal mortality, the highest risk of stillbirth was in the households in the rich category compared to the richest; AOR: 6.40 (95% CI: 3.69–11.11). Stillbirth was greater among households far from motorable road (≥6 km) compared to those at a distance of 5 km to the road; AOR: 3.40 (95% CI: 1.91–6.06), and greater in households that had three or more births in five years compared to two or fewer births; adjusted OR: 4.55 (95% CI: 3.35–6.16).

**Table 6:** Factors associated with stillbirths, south-west Ethiopia 2006–2010.

Predictors	groups	Stillbirths in household? (n = 6572 HH^A^)		Crude		Adjusted^E^	
		Yes	No	OR	95% CI	OR	95% CI
Wealth^B^	Richest 25%	16	1337	Ref		Ref	
	Rich 25%	106	1703	5.20	3.12, 9.11	6.40	3.69, 11.11
	Poor 25%	35	1673	1.75	0.97, 3.25	3.28	1.76, 6.11
	Poorest 25%	32	1551	1.72	0.95, 3.23	3.13	1.66, 5.87
Education^C^	Higher (9th+)	5	274	Ref		Ref	
	Primary (1–8)	52	2394	1.19	0.50, 3.40	1.03	0.40, 2.65
	Illiterate^D^	134	3708	1.98	0.87, 5.52	1.25	0.50, 3.16
Distance to road	≤5 km	14	1610	Ref		Ref	
	≥6 km	178	4770	4.29	2.48, 7.42	3.40,	1.91, 6.06
No. of births^F^	≤2	107	5451	Ref		Ref	
	≥3	85	929	4.66	3.48, 6.25	4.55	3.35, 6.16

A HH =  households (because there were more than one such events in some households, the number of households having stillbirths are different from the number of stillbirths; 226 stillbirths in 192 households).

B 119 households, including 3 with stillbirths, have missing value on the wealth index due to incomplete asset variables.

C Education of head of household ^D^Illiterate  =  cannot read and write ^E^adjusted to the other variables in the table ^F^number of births in five years.

Note: Hosmer-Lemeshow Test of Model fit: X^2^ (df) = 7.98 (7); p = 0.33. A p-value greater than 0.05 shows that the model well fit the data.

### Clustering of mortality in similar households

Table 7 presents the concentration of maternal and newborn mortality in certain households. Of the 49 households that had maternal deaths, nearly half (46.9%) also experienced either a stillbirth or neonatal death; 12 (24.5%) had stillbirths, and 11 (22.4%) had neonatal deaths. The likelihood of neonatal death in households that had maternal deaths was seven times higher compared to households that had no maternal mortality; OR: 7.2 (95% CI: 3.6–14.3). The similar likelihood of having a stillbirth in households that had maternal mortality was 11 times greater compared to households without maternal death; OR: 11.6 (95% CI: 6.0–22.7).

**Table 7:** Clustering of maternal and neonatal mortality and stillbirth in households, south-west Ethiopia 2006–2010.

		Maternal death in the household?^X^		OR (95% CI)
		Yes (n = 49)	No (n = 6523)	
Stillbirths in household?	yes	12 (24.5%)	180 (2.8%)	11.6 (6.0, 22.7)
	No	37 (75.5%)	6343 (97.2%)	Ref
Neonatal deaths in household?	yes	11(22.5%)	254 (3.9%)	7.2 (3.6, 14.3)
	No	38 (77.5%)	6269 (96.1%)	Ref

Note: ^X^Maternal mortality in a household was considered an exposure variable for stillbirth and neonatal death outcomes.

## Discussion

In this household study we found a maternal mortality ratio of 425 per 100,000 live births, a neonatal mortality ratio of 27 per 1,000 live births, and a stillbirth rate of 19 per 1,000 births. The risk of neonatal mortality was associated with the wealth status of the households, literacy status of the head, non-spaced births, and the distance from motorable road of households. The risk of stillbirth was also associated with the wealth, distance of the household from motorable road, and non-spaced births. The maternal mortality was also high among the poorest households compared to the richest and households that had maternal mortality also experienced a clustering of neonatal mortality or stillbirths. Moreover, the institutional delivery rate was unacceptably low.

To the best of our knowledge, this study is the first to describe three mortality estimates and associated household-risk factors using a large sample with high response rate in Gamo Gofa. In fact, there is a limited amount of evidence of maternal and neonatal mortality and stillbirths using community-based data from southern Ethiopia [14]. We used data collectors who had experience and were sensitive to the cultural taboos of the respective villages where they collected the data. Being the residents in their villages, they participated in all social events, including involvements in vital events such as celebrating births, caring for the sick, and funerals for the dead. The experience and deep knowledge of the area enabled them with the skill to handle sensitive questions and recall many of the deaths that occurred in their villages. We also used an experienced nurse as a field technical supervisor to help in classifying deaths by using the verbal autopsy method.

Because of lack of previous reports from the area, we compare our findings with community-based studies from other provinces and the national level estimates for Ethiopia. As a result, we do not know whether our findings were under-reported or represent the reality of the area. The maternal mortality ratio of 425 per 100,000 live births was similar to the findings of community-based studies decades ago in other parts of Ethiopia: the MMR per 100,000 live births was 402 in Jimma in 1990 [23], and 440 in Butajira in 1996 [24]. However, our estimate is higher than the UN and World Bank's estimate for Ethiopia of 350 per 100,000 live births in 2010 [1]. If we adjust our maternal mortality estimate upward by a factor of 1.6, it yields an MMR of 680 per 100,000 live births. The 2011 DHS reported MMR of 676 per 100,000 live births for Ethiopia [25] which is similar to our upwardly adjusted estimate. The suggestion as well as the factor of adjustment were conducted according to the recommendation by Santon and colleagues for the correction of the potential under-reporting of demographic studies [26]. The under-reporting of maternal deaths is a well-recognized global problem [27]–[29], and our study may not have escaped the challenge. However, given a general downward trend of Ethiopia's MMR estimate by the UN inter-agency [1], and Hogan et al [30], our direct (unadjusted) estimate of an MMR of 425 per 100,000 may be close to reality.

Our neonatal mortality estimate of 27 per 1,000 live births is lower than the DHS national estimate of 37 [31], and the UN Inter-agency estimate of 31 for Ethiopia in 2011 [20]. However, since mathematically modeled UN estimations often have inconsistencies with survey findings, and we have limited locally available estimates, we cannot judge whether our finding is due to under-reporting. Regarding stillbirths, Cousens et al estimated Ethiopia's stillbirth rate as 26 per 1,000 births in 2009 [9], which is still higher than our finding of 19 per 1,000 births. In the ethnic “Gamo” culture, a birth of a dead fetus and the early death of a newborn are not publicized and not publicly mourned. Only close relatives and family members are informed and other people are told the incident as “something went wrong” in local terms. This can make it difficult for an outsider to distinguish between a neonatal death and a stillbirth. Accordingly, though we tried to minimize under-reporting by carefully choosing the data collectors, some of the neonatal deaths and stillbirths may have been missed in this study. Global estimates suggest that 75% of neonatal deaths take place in the first week of life [4]. In our finding, first-week deaths were approximately half of the neonatal deaths (46.4%) which may also suggest an under-reporting of early neonatal deaths and stillbirths.

The present study showed that indicators of the socio-economic status (SES) of households (wealth, education of head of household, the distance of households from a motorable road), and a factor related to reproductive health (non-spaced births) were associated with stillbirths and neonatal mortality outcomes. We examined the effect of household wealth (poor-rich differences) on mortality outcomes and found a significant variation. The poorest households were more likely to have mortality outcomes compared to the richest. Nevertheless, when compared to the richest quartile, the greatest risk of both neonatal mortality and stillbirth was to the rich households, not to the poorest.

Households in the poorest category were the second most at-risk group for the mortality outcomes. This finding needs further investigation with a focus on how the true wealth status can be determined at the particular stage of socio-economic development in the rural Ethiopia [21]. Our opinion is that the asset variables selected to indicate wealth status may not have correctly reflected the actual wealth status of all households. For example, farming land, cattle, and crops may not be owned by young people who returned to rural residence after certain years of education in urban areas. In addition, educated residents such as government employees who usually do not have these rural assets may have been wrongly classified as poor. Our finding of the association of education with neonatal mortality could also support the idea that the educated, but classified as poor and poorest in terms of rural wealth, may have been relatively better-off compared to those in the rich category because of the knowledge advantage.

On the other hand, households in the richest wealth category had the lowest risk of mortality possibly because of their economic access to health services and expected better living conditions. In general, the findings that the richest households had advantages while households in the three other categories experience greater risk could be due to the situation of wealth where few households have greater possession of assets and the other majority is homogeneously poor. The finding agrees with a previous analysis of Ethiopian rural asset data that classified the majority (up to 60%) of households as having low SES, thereby suggesting a homogeneity of most households in asset ownership [21].

Economists measure economic status indicators through information from income or expenditure, which is difficult to gather in low-income countries, and asset-based wealth is an alternative proxy in less developed areas [32]. Practically speaking, the wealth index is equally valid to income or expenditure data for health surveys in Africa [33], and the effect of household wealth on health outcome is well known [34]–[36]. Less clear, however, is how wealth causes mortality difference in areas where the overall access to health services and service utilization is very low to all people such as those in our study area. For instance, in our results, there is the poorest-richest difference in maternal mortality and stillbirths. Even so, very few households (including the richest) utilize health institutions for delivery service. This indicates that household wealth contributes to maternal mortality in mechanisms other than those that create economic access to health service. These mechanisms may include, e.g., improved, clean housing and better nutrition [37]. Unfortunately, this is beyond the aim and limitations of our study.

Regarding the association of wealth with neonatal mortality, the poorest-richest difference may be due to economic access to antibiotics and other drugs from rural private venders, where a wealthy family often has a better access in addition to better nutrition and improved housing since access to antibiotics plays an important role in newborn survival [38]. In addition, the association of neonatal mortality with the education of the heads of a households and the distance to a motorable road further suggests the importance of these variables as tools to access health interventions. The education of parents has a positive correlation with better health of children through better knowledge of solutions and critical decisions during crisis, in addition to the opportunity education creates for job and economic access [39]–[42]. However, in the rural area where this study took place, few people had achieved better jobs despite higher levels of education. The knowledge advantage may have played a role related to access and utilization of treatments in households with educated heads.

Our study also demonstrated an association of household distance from a motorable road with neonatal death. Travel distance is clearly an important factor once the decision is made to seek medical care during critical conditions and distance to a motorable road has dual effects: 1) as a disincentive to seek health care, and 2) as a barrier to reach the relevant facility [43]. As such, people in households closer to a motorable-road are more likely to seek health care and save lives. We also found an increased likelihood of neonatal mortality and stillbirths among households where there was a maternal death. This is in agreement with a finding in a WHO multi-country maternal and newborn health survey, which showed a seven fold greater early neonatal mortality, in which mothers had died or developed nearmis (dangerous illness) compared to mothers without these events [44]. The clustering of maternal and neonatal mortality, as well as stillbirths in certain households, illustrates how impoverished households are trapped in many adverse outcomes.

The findings of a low utilization of facilities for skilled delivery, compounded with high mortality rates, call for educating mothers and other family members the importance of seeking skilled delivery service (institutional deliveries). Furthermore, improving the quality of the existing poorly equipped health institutions in the area [45] might help to increase the willingness and trust of families to utilize these institutions. The Ethiopian health extension workers have the opportunity to educate women and family, as they have close contact during the antenatal care and the routine household visits. In summary, our findings highlighted that households living in the villages far from road access, poor SES, and having illiterate household heads and non-spaced births, had greater risks of mortality. Therefore, a targeted follow-up of pregnancies in these households could help to achieve reduced mortality outcomes. Interventions such as family planning education and the availability of FP technology choices to support women in poor households may help prevent deaths caused due to the risk of non-spaced births.

In order to obtain ongoing data to help monitor progress, the community-based registration of pregnancy and birth outcomes (abortions, stillbirths, livebirths, neonatal and maternal deaths) may be the ideal option. In addition to providing sustainable data for evaluation of effectiveness of policy and programmes, ongoing registration can provide evidence for rights- based advocacy for improvement of health services, and many other benefits outside of the health sector [46]. For prospective registration to occur, Ethiopia must utilize the privilege of the available two health extension workers (HEWs) responsible for each kebele (average 500 households per HEW). Registration-based information may have benefits of reduced risk of recall bias and the cost of surveys by using the already available community health workers for active data collection.

In the following, we address some of the limitations of the study. First, recall bias and under-reporting are widely recognized problems in studies that ask respondents about past events. The intensity of the bias depends on the time interval between the event and the sensitivity of the event to memory [3]. We tried to reduce recall bias in two ways: 1) by selecting data collectors from the respective villages of the data collection that the data collectors helped the respondents to recall the events through their in depth knowledge of social events that happened in their villages, and 2) by choosing a memorable and short time reference period for the event to be recalled. However, there might have been some deaths that were missed due to recall bias in the current data. Second, as it is well-known in survey studies, we cannot show the temporality (time sequence) of the occurrence of exposures and outcomes [47]. For example, having more than three births in the last five years in households was associated with both neonatal mortality and stillbirth. Nevertheless, we cannot assure whether neonatal mortality and stillbirths led to more births or whether more births led to a greater risk.

Third, we used reported information from family members on mortality outcomes and mid-level expert decisions of the cause-of-death classifications using a simplified verbal autopsy technique, which also has the potential for misclassification (misdiagnosis). The existence of misclassification in using the verbal autopsy was reported from a well-designed prospective study of maternal deaths in Guinea-Bissau in Africa as 30% of maternal deaths were left unclassified [48]. The confirmatory diagnostic method used to ascertain the cause of maternal death is an autopsy test which has been used in a hospital setting in Mozambique in Africa [49]. However, such a modern technology cannot be applied in a rural community such as Bonke. Fourth, we were not able to show yearly changes in mortality. We aimed to describe at least aggregated measures by using a single reference period in a community where the date of the event is not easy to identify. From a practical point, it is difficult and inappropriate to expect a specific time-related response from a largely illiterate rural society where there is no vital registration system.

## Conclusions

Mortality rates are still lagging behind the MDG targets for Ethiopia. There also exist socio-economic inequalities in maternal and neonatal mortalities, as well as stillbirths in the area. The socio-economic inequality in mortality and the low utilization of existing institutions for delivery care highlight the importance of quality emergency obstetric care service. The services need to target the poorest households where mortalities cluster and disproportionately high. It is important to address barriers to accessing institutional delivery services in a way that is acceptable for rural women.

## Supporting Information

File S1 **Supporting information file (two tables informing variables included in Principal Component Analysis (PCA).** Table S1. Variables included in Principal Component Analysis (PCA) for wealth index creation. Table S2. Background descriptions of the variables included in the PCA analysis.
(DOCX)
