# Supplementary material for: Maternal and Neonatal Mortality in South-West Ethiopia: Estimates and Socio-Economic Inequality
Source: PLoS One. 2014 Apr 30;9(4):e96294. doi: 10.1371/journal.pone.0096294 (PMC4005746; doi:10.1371/journal.pone.0096294)
Supplement: File S1 — Supporting information file (two tables informing variables included in Principal Component Analysis (PCA). Table S1. Variables included in Principal Component Analysis (PCA) for wealth index creation. Table S2. Background descriptions of the variables included in the PCA analysis. (DOCX) [file pone.0096294.s001.docx]

| Variable | Value allocation | |
| --- | --- | --- |
| 1 )Cattle owned | No=0 | Yes=1 |
| 2) Farm-land available | No=0 | Yes= 1 |
| 3) Horse/mule owned | No=0 | Yes=1 |
| 4) Cash crop producer? | Not=0 | Yes=1 |
| 5) Goat/sheep owned? | No=0 | Yes=1 |
| 6) Bed and table present? | No=0 | Yes=1 |
| 7) Radio owned? | No=0 | Yes=1 |
| 8) Kitchen separate? | In living house=0 | Separate=1 |
| 9) Source of drinking water | Unprotected source=0 | Pumped, protected spring, protected well=1 |
| 10) Type of latrine | Pit or no latrine=0 | All other improved=1 |

### Table S1:

Table S2:

| Variables (n=6572 HHs) | Mean score | SD | Communalities | Correlation with the first component |
| --- | --- | --- | --- | --- |
| 1 )Cattle owned? | 0.93 | 0.24 | 0.54 | 0.50 |
| 2) Farm-land available? | 0.95 | 0.21 | 0.46 | 0.49 |
| 3) Horse/mule owned? | 0.28 | 0.45 | 0.34 | 0.58 |
| 4) Cash crop producer? | 0.31 | 0.46 | 0.27 | 0.35 |
| 5) Goat/sheep owned? | 0.69 | 0.46 | 0.36 | 0.59 |
| 6) Bed and table present? | 0.33 | 0.47 | 0.44 | 0.45 |
| 7) Radio owned? | 0.13 | 0.34 | 0.44 | 0.45 |
| 8) Kitchen separate? | 0.38 | 0.48 | 0.18 | 0.37 |
| 9) Source of drinking water? | 0.13 | 0.34 | 0.06 | 0.23 |
| 10) Type of latrine? | 0.21 | 0.41 | 0.50 | 0.41 |
| **Note**: Eigen value= 2.06 means the variability explained by the main (first) component= 20.6% | | | | |
